# Estimation of Emotional Arousal Changes of a Group of Individuals During Movie Screening Using Steady-State Visual-Evoked Potential

**DOI:** 10.3389/fninf.2021.731236

**Published:** 2021-09-02

**Authors:** Seonghun Park, Do-Won Kim, Chang-Hee Han, Chang-Hwan Im

**Affiliations:** ^1^Computational Neuroengineering Laboratory, Department of Biomedical Engineering, Hanyang University, Seoul, South Korea; ^2^Department of Electronic Engineering, Hanyang University, Seoul, South Korea; ^3^Department of Biomedical Engineering, Chonnam National University, Yeosu, South Korea; ^4^School of Healthcare and Biomedical Engineering, Chonnam National University, Yeosu, South Korea; ^5^Department of Software Engineering, Dongseo University, Pusan, South Korea

**Keywords:** electroencephalography, SSVEP, neurocinematics, emotion estimation in crowds, passive brain-computer interface

## Abstract

Neurocinematics is an emerging discipline in neuroscience, which aims to provide new filmmaking techniques by analyzing the brain activities of a group of audiences. Several neurocinematics studies attempted to track temporal changes in mental states during movie screening; however, it is still needed to develop efficient and robust electroencephalography (EEG) features for tracking brain states precisely over a long period. This study proposes a novel method for estimating emotional arousal changes in a group of individuals during movie screening by employing steady-state visual evoked potential (SSVEP), which is a widely used EEG response elicited by the presentation of periodic visual stimuli. Previous studies have reported that the emotional arousal of each individual modulates the strength of SSVEP responses. Based on this phenomenon, movie clips were superimposed on a background, eliciting an SSVEP response with a specific frequency. Two emotionally arousing movie clips were presented to six healthy male participants, while EEG signals were recorded from the occipital channels. We then investigated whether the movie scenes that elicited higher SSVEP responses coincided well with those rated as the most impressive scenes by 37 viewers in a separate experimental session. Our results showed that the SSVEP response averaged across six participants could accurately predict the overall impressiveness of each movie, evaluated with a much larger group of individuals.

## Introduction

Neurocinematics is an emerging interdisciplinary research field that aims to provide a new method to quantitatively evaluate films or movie contents by employing neuroscientific techniques to analyze viewers’ brain activities during movie screening (Hasson et al., 2008). In the earliest neurocinematics study, participants lay on their backs in a magnetic resonance imaging (MRI) scanner and watched short movie clips, while their brain activities were recorded using functional MRI (fMRI) (Hasson et al., 2004). In their study, inter-subject correlation (ISC) was implemented to measure the inter-subject synchronization of cortical activities. The ISC index effectively indicated the similarity between the viewers’ brain activities and the response to the presented movie content. Additionally, the authors demonstrated the feasibility of using the ISC index to assess the impact of different filmmaking styles on viewers’ brains. Since the first study, most neurocinematics studies have been conducted based on fMRI (Naci et al., 2014; Kauttonen et al., 2015; di Oleggio Castello et al., 2020).

Although fMRI-based neurocinematics studies have shown promising results and new perspectives, these studies pose numerous limitations for use in practical applications (Dmochowski et al., 2012; Lankinen et al., 2014; Cha et al., 2015). For example, the accessibility to fMRI is limited owing to its high cost and poor portability. Moreover, the experimental environments are different from those for general film screening, that is, the study participants should lie on their back in a narrow MRI scanner while watching a small-sized screen and enduring unpleasant scanning noises. To address this issue, Dmochowski et al. employed electroencephalography (EEG) as a new modality to record viewers’ brain activities (Dmochowski et al., 2012). They extracted maximally correlated signal components from multiple EEG channels and then compared the extracted components with movie scenes. They proposed two indices, intra-subject correlations (IaSC) and ISC, to estimate the engagement of viewers in video clips. They found that the peaks of IaSC were observed during highly arousing scenes, and the ISC value decreased when the viewers watched the same video again. However, their method still had a limitation in that it required recording EEG signals from many scalp electrodes, preferably covering the entire scalp area, to reliably evaluate the indices. Moreover, because these indices were based on a data-driven approach without a well-established hypothesis, they can be readily dependent on a particular group of participants.

In this study, we propose a novel method to continuously track temporal changes in emotional arousal during movie screening using only three electrodes. We employed a characteristic brain response known as steady-state visual evoked potential (SSVEP), a train of periodic EEG waves elicited by visual stimuli flickering or reversing at a specific frequency. SSVEP has been widely used in brain-computer interface (BCI) applications since the early days of BCI studies (Regan, 1977; Cheng et al., 2002). SSVEP responses can be readily recorded from electrodes attached to the occipital region where the primary visual cortex is located (Regan, 1966). Accordingly, the number of EEG electrodes can be minimized. Because of the periodic nature of the SSVEP responses, the spectral power at the flickering frequency of visual stimuli remains stable over time, which makes SSVEP useful in many applications with high robustness to noise (Vialatte et al., 2010). Moreover, the amplitude of the SSVEP has been reported to be modulated by the individual subject’s mental states, such as attention (Di Russo et al., 2003), cognitive load (Wu et al., 2010), and emotional arousal (Keil et al., 2003). Based on these characteristics of SSVEP, two emotionally arousing movie clips were superimposed on a background eliciting an SSVEP response at a specific frequency. We then continuously monitored the changes in the SSVEP responses of a small group of participants during the movie screening, based on the hypothesis that changes in emotional arousal can affect changes in the amplitude of the SSVEP responses. We then investigated whether the movie scenes that elicited higher SSVEP responses in the small group of participants matched those rated as the most impressive by a much larger group of people who watched the movie clip in a separate experimental session.

## Materials and Methods

### Participants

Six healthy right-handed adults with normal or corrected-to-normal vision served as paid volunteer participants. All participants were adult males, with an average age of 22.3 ± 1.7 years. None of the participants had a history of neurological or psychiatric disease. Following a thorough explanation of the study protocol, all participants signed an informed consent form. All experimental protocols were approved by the institutional review board of Hanyang University (IRB No. HYI-14-167-11) according to the Declaration of Helsinki.

### Emotionally Arousing Movies

Two 5-min video clips were extracted from emotionally arousing movies: “Ju-on 2” (2003) and “Bang! You’re Dead” (1985) to elicit fearful and nervous emotions, respectively. First, “Ju-on 2,” a world-renowned Japanese horror film, was edited to include both scary and non-scary scenes for this experiment. To maximize the fearful emotions of the viewers, the scary scenes included the sudden appearance of specters with thrilling background music. Non-scary scenes, mostly consisting of everyday scenes that do not contain any specter or thrilling background music, played a role in bridging the events required to form a complete story. “Bang! You’re Dead” was a famous TV series of a film director Alfred Hitchcock, which has been employed in previous neurocinematics studies (Hasson et al., 2008; Dmochowski et al., 2012). The episode employed in our experiment was about a boy who mistook a loaded revolver as a toy gun. The movie was edited to contain both nervous and non-nervous scenes. The nervous scenes displayed the boy loading the revolver or pulling the revolver trigger, whereas the non-nervous scenes did not contain any nervous events. Each frame of the movie clips was adjusted to maintain a constant luminance throughout the screening time using a custom script written in MATLAB (Mathworks, Inc., Natick, MA, United States) to prevent the SSVEP amplitude from being modulated by luminance change (Floriano et al., 2018).

### SSVEP Stimulus

**FIGURE 1:**
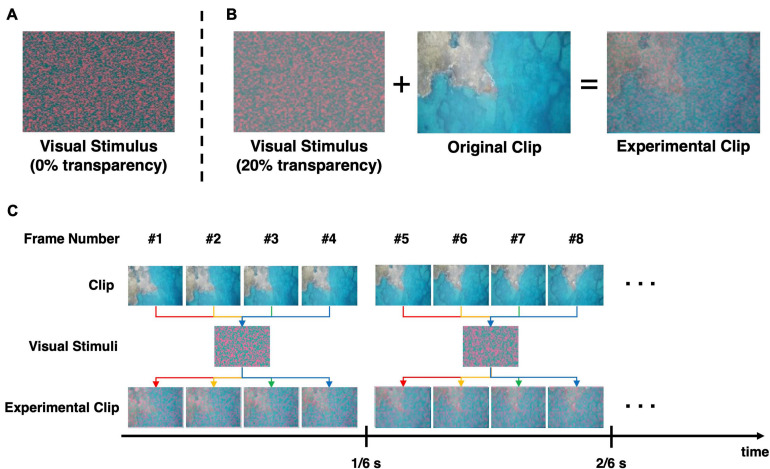
Procedure to generate the experimental videos employed in this study. **(A)** The chromatic random-noise-like visual stimulus. **(B)** The conceptual procedure of generating the experimental clip. The visual stimulus was applied with 20% transparency and then superimposed on the original clip. **(C)** The overlaying process to create flickering effect for evocation of SSVEP response. The visual stimulus that was overlapped on the clip changed every 1/6 s, so that the resultant video flicker at 6 Hz. Note that the frame rate of the experimental video was 24 frame/s, and the same visual stimulus was maintained for subsequent four frames.

In our experiments, a new visual stimulus was used to continuously evoke the SSVEP response at a specific frequency, while the participants watched the designated video clips. The visual stimulus consisted of small-sized colored dots randomly scattered to cover the entire screen. The size of each dot was set as small as possible (single pixels in this study) to minimize the distraction of the viewers. Because the red/green-colored chromatic visual stimulus was reported to cause less eye fatigue than the conventional white/black-colored stimulus (Lai et al., 2011) with a comparable amplitude of SSVEP responses being evoked (Lim et al., 2017), the red/green-colored dots were scattered over the screen, as shown in Figure 1A. Six still images of different patterns of scattered dots were generated. Then, a 20% transparency was applied to the six visual stimuli so that the movie contents could be readily identified after the original movies were overlaid on the visual stimuli (Figure 1B). Finally, to evoke SSVEP responses at 6 Hz, six still images of different patterns of scattered dots were combined into a background video, with a frame rate of six frames per second. Because the frame rate of the original movie clips was four times higher than that of the background video, four frames of the original clip were superimposed with one frame of the background video, as depicted in Figure 1C. This video clips were edited using Adobe Premiere CS6 (Adobe Inc., San Jose, CA, United States). The edited video clips can be found on YouTube^TM^ with the following link: https://youtu.be/xeqwAVO0UFg (Note: it is not recommended to watch the movie clips if the reader does not like horror movies).

### Experimental Paradigm

The participants sat on a comfortable armchair and watched the clips presented on a 24″ LCD monitor screen 70 cm away from them. The EEG data were recorded while the participants watched the clips, and they reported the two most emotionally impressive (e.g., most fearful or most nervous) scenes after watching each video clip. The participants were instructed to select two scenes in each video clip because the participants could have difficulty selecting more than two impressive scenes in such a short video. The order of movie screening was randomized across participants. Additional 31 participants of similar age and gender were also presented with the two video clips without EEG recording. The weblinks to the two video clips were provided to them; then, each participant responded to the two most emotionally impressive scenes in each movie clip through an online poll.

### Data Recording and Analysis

The EEG data were recorded at a 2,048 Hz sampling rate from three scalp electrodes (O1, Oz, and O2) using a commercial biosignal recording system (BioSemi ActiveTwo, Biosemi, Amsterdam, Netherlands). The ground and reference electrodes were replaced with a common mode sense active electrode and a driven right leg passive electrode, both of which were located in the posterior region. The recorded EEG data were down-sampled to 512 Hz and re-referenced to Cz. Since we only analyzed the changes of the SSVEP response at the stimulation frequency, no other artifact elimination techniques were applied. The temporal changes in the spectral power at 6 Hz were evaluated using fast Fourier transform (FFT) with a sliding 10-s moving window and a 90% overlap. Then, the spectral power series was z-score normalized and grand averaged over the O1, Oz, and O2 channels and all experimental participants.

**FIGURE 2:**
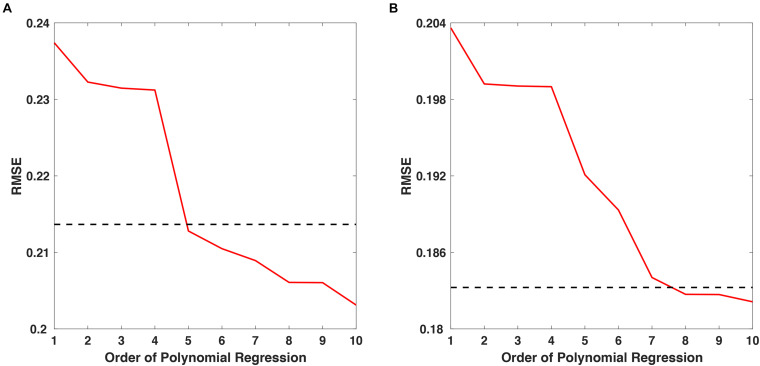
The RMSE changes as a function of polynomial order for approximating SSVEP responses for **(A)** “fear” and **(B)** “nervous” videos. The dashed line indicates 0.9α, where α is the RMSE of the 1st-order polynomial regression.

Polynomial regression was employed to estimate the overall trend of temporal changes in spectral power from small-sized samples. The order of the polynomial regression was determined by finding the lowest order in which the root-mean-square error (RMSE) of the polynomial regression reduced to less than 0.9 α, when α indicates the RMSE of 1st-order polynomial regression. Figure 2 shows the RMSE changes as a function of the polynomial order with respect to the “fear” and “nervous” videos, with dashed lines indicating the RMSE of 0.9 α for each video. For the “fear” video data, the lowest order that showed an RMSE less than 0.9 α was 5 (Figure 2A), while the lowest order that showed an RMSE less than 0.9 α was 8 for the “nervous” video data (Figure 2B). Therefore, the regression models with 5th- and 8th-order polynomials were employed for approximating SSVEP responses for “fear” and “nervous” videos, respectively.

Lastly, the regression results were qualitatively and quantitatively compared with the survey results; the survey consisted of questions regarding the most impressive scenes. To compare the regression results with the survey results, a moving average with a window size of three samples was applied to the survey results. Then, to evaluate the correlations between the regression results and survey results, we computed Pearson’s correlation coefficient. Because SSVEP regression results had a total of 270 data points while the survey results had only 27 data points, we averaged every 10 SSVEP regression results to match the number of data points in both data. Please note that moving average was not applied to the regression results of SSVEP response.

## Results

### Validation of SSVEP Evocation

**FIGURE 3:**
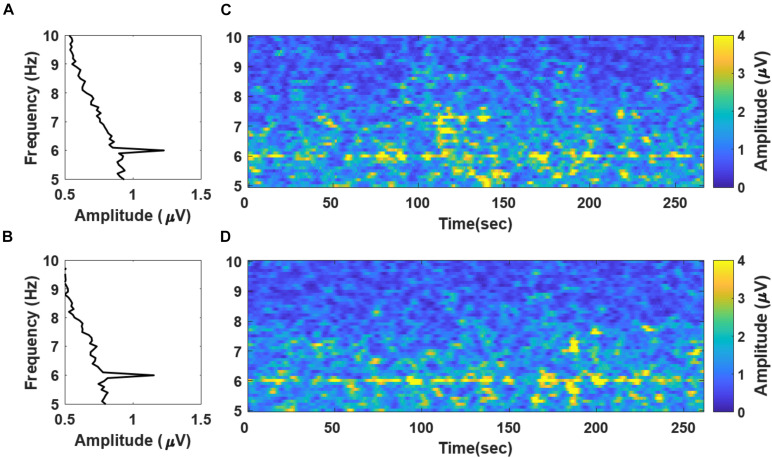
Grand averaged frequency spectra and spectrograms evaluated with the EEG data recorded during **(A)** “fear” and **(B)** “nervous” movie screenings. A notable peak of SSVEP component is observed at 6 Hz. The spectrograms evaluated with 10 s moving window with 90% overlap of “fear” and “nervous” videos are presented in panels **(C,D)**, respectively. Please note that the frequency spectra in panels **(A,B)** are presented in a “90°-rotation” manner for matching with the y-axis in the spectrogram in the right panels **(C,D)**.

To validate whether the SSVEP was successfully evoked by the newly proposed stimuli, we calculated the grand averaged spectral power of the recorded EEG data during “fear” and “nervous” movie screening (Figures 3A,B, respectively). The frequency spectra demonstrate distinct peaks at the stimulation frequency of 6 Hz. Figures 3C,D show the spectrogram of the grand averaged temporal changes in spectral power during movie screening with respect to “fear” and “nervous” clips, respectively. The spectrograms depicted that the spectral power at the stimulation frequency was distinctly higher than that at the remaining frequency bands in both clips. Based on the results, we confirmed that the SSVEP response was successfully evoked by the new chromatic random-noise-like stimuli during the movie screening.

### Prediction of the Viewers’ Response

**FIGURE 4:**
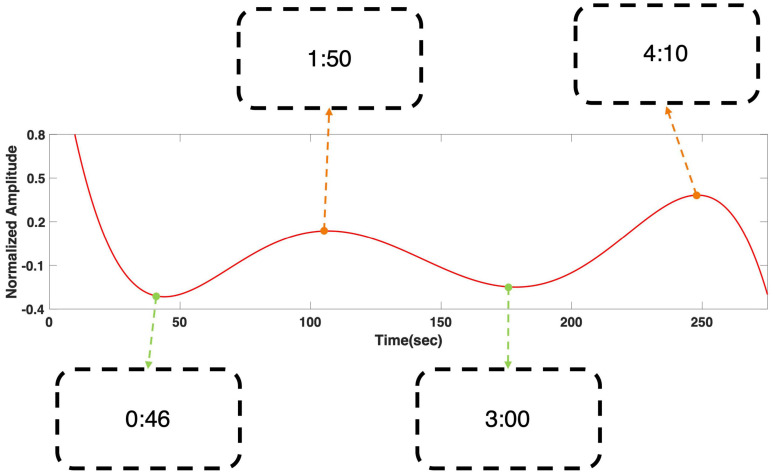
Grand averaged polynomial regression result of SSVEP response at 6 Hz during “fear” movie screening. The minima corresponded with relatively ordinary scenes, while the maxima corresponded to the scenes with appearance of specter. The scenes corresponding to each local extrema can be found using the time stamps in the boxes. The time stamps correspond to the time points in the following movie clip: https://youtu.be/xeqwAVO0UFg.

**FIGURE 5:**
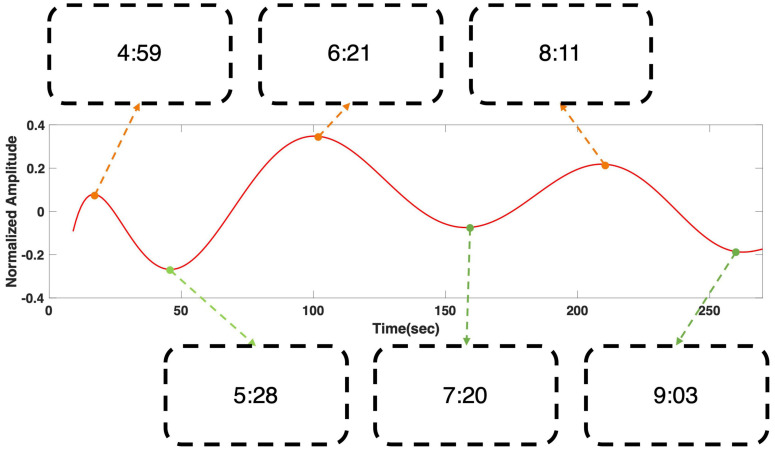
Grand averaged polynomial regression result of SSVEP response at 6 Hz during “nervous” movie screening. The minima corresponded to the scenes that play a role in connecting the events to proceed the story, while the maxima corresponded to the scenes in which a boy was loading or triggering a gun. The scenes corresponding to each local extrema can be found using the time stamps in the boxes. The time stamps correspond to the time points in the following movie clip: https://youtu.be/xeqwAVO0UFg.

Figure 4 shows the regression result of grand averaged temporal power changes at 6 Hz during “fear” movie screening. It shows four local extrema, where the gradient of the graph became zero, and the time stamps of the scenes that corresponded to each local extremum were presented. The time stamps in the boxes correspond to the time points in the following movie clip: https://youtu.be/xeqwAVO0UFg. While the maxima were observed in scenes with specters and thrilling background music, the minima were observed in the scenes with relatively ordinary daily life events without specters. Figure 5 shows the regression result of grand averaged temporal power changes at 6 Hz during “nervous” movie screening, which showed three local maxima and three local minima, similarly to the “fear” movie screening. In the comparison of each extremum with the corresponding movie scene, maxima were observed in scenes where the revolver was loaded or triggered. In contrast, the minima were observed in scenes that played a role in progressing the story, which did not include scenes of loading or triggering the gun.

**FIGURE 6:**
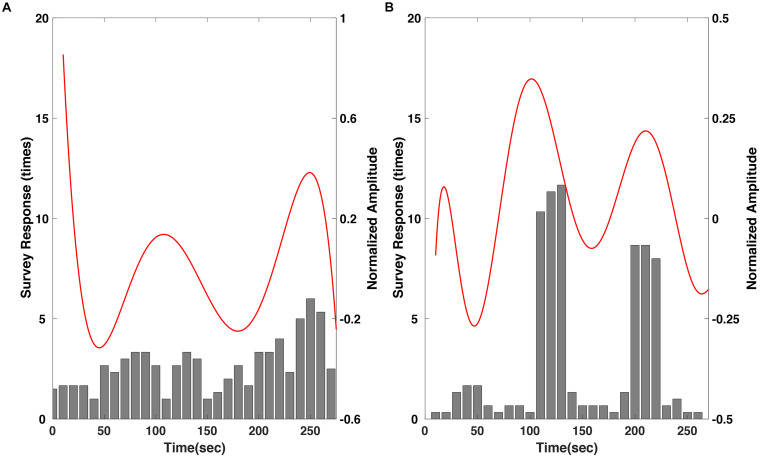
Comparison of the polynomial regression results of SSVEP response to the survey results of the most emotionally arousing scenes in the clips, for the **(A)** “fear” and **(B)** “nervous” videos. Please note that the survey results were moving averaged with a window size of three samples.

Then, the regression results were compared with the survey results of most impressive scenes with respect to each movie to evaluate whether the response of the larger group can be predicted using the proposed method (Figure 6). In the “fear” video, the survey results showed a tendency to slowly increase over time throughout the video (Figure 6A). This indicates that the video induced more emotional arousal as it progressed, and the regression result corresponded well with this tendency. In particular, the number of votes was highest near the end of the video, approximately 250 s, where the amplitude of the regression results also showed the largest local maximum value. The correlation coefficient between the regression results and the moving averaged survey results was 0.49 with a *p*-value of 0.0117 (*p* < 0.05). Meanwhile, in the “nervous” video, the survey result was remarkably distinct; the reports were mostly focused in two scenes; (i) The boy loaded the revolver; (ii) The boy triggered the loaded gun (Figure 6B). The regression results also corresponded well with the survey result, showing distinct local maxima at the time stamps corresponding to the two scenes. Interestingly, the regression results showed a total of three maxima, while the survey results focused on two scenes. This could be because the participants were instructed to select only two most arousing scenes. The correlation coefficient between the regression results and the moving averaged survey results was 0.41 with a *p*-value of 0.0382 (*p* < 0.05). The raw EEG datasets and survey results are available at the following link: https://figshare.com/s/745412a9047b478c3f81.

## Discussion

In this study, we proposed a new method for estimating emotional arousal changes in a group of individuals during video screening by employing a novel visual stimulation method that overlays a background SSVEP stimulus on a video clip. We first confirmed that the SSVEP response was stably evoked by the proposed visual stimulus throughout the screening of the movie clip (Figure 3). Because the proposed method was based on the modulation of SSVEP amplitude, the steady evocation of SSVEP response throughout the entire experimental period was essential. Then, we confirmed that the regression results were in a good accordance with the survey results acquired from 37 viewers, both qualitatively and quantitatively (Figure 6). The correlation coefficients between the regression results and the moving averaged survey results were 0.49 with a *p*-value of 0.0117 (*p* < 0.05) for the “fear” video, and 0.41 with a *p*-value of 0.0382 (*p* < 0.05) for the “nervous” video. In addition, to further verify the effectiveness of the proposed method, we compared the amplitude changes and the time stamps of the regression results with the corresponding scenes of the clip, as in the previous literatures (Wagner et al., 2011; Dmochowski et al., 2012; Han et al., 2017). It was also confirmed that the polynomial regression results of the SSVEP response were qualitatively in good accordance with arousing scenes of the movie clips (Figures 4, 5). As reported in the previous SSVEP studies (Keil et al., 2003, 2008), the SSVEP responses showed a tendency to increase on the time stamps of the scenes with arousing events while decrease on the time stamps of the scenes with relatively ordinary events. To the best of our knowledge, this is the first study that used SSVEP responses to track brain state changes in a group of viewers during video screening.

In the previous studies on the estimation of emotional state changes using physiological signals had some limitations. For example, Dmochowski et al. (2012) proposed a method to calculate ISC using EEG data acquired from 64 electrodes and compared the ISC changes during the movie screening with the corresponding scenes. However, the ISC is a data-driven method, which can be dependent on a particular group of participants. On the other hand, Brouwer et al. proposed a method to estimate the emotional arousal while reading an emotional novel, by combining various features evaluated from EEG, ECG, skin conductance, and respiration data (Brouwer et al., 2015). Also, Han et al. (2017) proposed a method to estimate the emotional arousal changes during movie screening, using an indicator called global field synchronization (GFS) calculated from 22 channel EEG data. However, these methods commonly required EEG data recorded from the whole head, and the method proposed by Brouwer et al. required even more numbers of modalities, limiting their use in practical scenarios. In contrast, the method proposed in the present study requires only three electrodes in the occipital region. In addition, we have demonstrated that only a small number of participants (six in this study) might be sufficient to estimate the grand-averaged emotional arousal changes of a larger group of participants, which were not fully verified in the previous studies.

In previous studies on SSVEP changes in response to brain state changes, different methods were implemented to evoke SSVEP responses. First, Bekhtereva et al. (2015) proposed 115 randomly distributed moving red squares superimposed upon a grayscale unpleasant or neutral emotional picture. To evoke an SSVEP response at 15 Hz, 35% of the dots moved coherently in one of the four directions (either up, down, left, or right) by 0.04° of visual angle for four frames, and the visual stimuli were presented with a screen having a refresh rate of 60 Hz. Similarly, Deweese et al. (2014) proposed 150 randomly distributed yellow dots superimposed upon a grayscale pleasant, neutral, or unpleasant emotional pictures. The dots flickered at a rate of 8.57 Hz to evoke an SSVEP response. The authors in the two abovementioned studies evaluated the SSVEP amplitude at the stimulation frequency and compared the amplitude difference between the emotion classes of each study. On the other hand, in a study by Hajcak et al., a picture containing emotional content (unpleasant or neutral) flickering at 15 Hz was presented (Hajcak et al., 2013). The authors evaluated the SSVEP response at 15 Hz then compared the SSVEP response difference between emotion classes. The proposed methods in the literatures successfully evoked the SSVEP response, however, such stimulation methods are inappropriate for video-type stimuli because video viewers dynamically change their focus during video screening, in contrast to static pictures. In this study, we proposed a novel visual stimulation method that presents many semi-transparent chromatic noise-like dots that are widely overlaid with the original video. Our stimulus presentation method seamlessly evoked an SSVEP response, regardless of the position of the screen the viewer was gazing at during video screening.

In the film editing stage, filmmakers must know whether the target emotion was successfully induced to the viewers as they had intended, because simply changing the sequence of some scenes of a film can significantly influence viewers’ emotional responses (Dmochowski et al., 2012; Cha et al., 2015). Therefore, the proposed method might be employed by filmmakers to evaluate the emotional arousal changes of a film. Furthermore, our experimental results showed good agreement with previous SSVEP studies that reported a positive correlation between SSVEP amplitude and emotional arousal (Keil et al., 2003, 2008). The proposed method can be specifically useful in evaluating viewers’ emotional arousal changes for emotionally arousing film genres, such as horror, action, and thriller movies. In contrast, our method might not be useful for evaluating viewers’ emotional changes for some film genres, such as comedy, documentary, and reality movies. Indeed, a study by Hasson et al. (2008) that used a data-driven method, ISC, also reported that 65 and 45% of the viewer’s cortex showed similar activities when thriller and action movies were presented, respectively; however, 18% and only a small fraction of the cortex (less than 5%) showed similar activities when comedy movies and reality videos were shown, respectively.

Recently, the decoding of emotional states from EEG has attracted increased attention because of the popularization of low-cost wearable EEG systems (Casson, 2019). Because SSVEP responses can be readily recorded with a few EEG channels covering a small portion of the occipital area, wearable EEG devices specialized for the evaluation of movies are expected to be relatively easily implemented. A recently developed wearable EEG device named NextMind^TM^^1^ might be an exemplar form factor for wearable devices based on our proposed method.

However, some potential issues exist that must be addressed before the proposed method can be used in practical applications. First, there are people who do not generate sufficiently large SSVEP responses even when identical flickering or reversing visual stimuli are presented (Kim et al., 2019). Second, viewers might feel uncomfortable because of the flickering dots on the background. Moreover, the superposition of the flickering dots on the video might affect the emotional arousal of the viewers. While there are a number of studies that successfully induced emotional states to the participants while they were presented with a visual content superposed under a flickering stimulus (Müller et al., 2008; Deweese et al., 2014), the impact of the proposed visual stimulus presentation method on the emotional arousal of the viewer’s needs to be further investigated.

In this study, all subjects who participated in the EEG recording experiment were males and the number of the participants was relatively small (only six in this study), because some participants that we had recruited were excluded as they did not exhibit clear SSVEP response peak at 6 Hz. In our experiments, there were more than six participants (eight participants) whose EEG signals did not exhibit clear SSVEP peaks particularly because our background visual stimuli were too weak to evoke SSVEP responses in some participants. Nevertheless, we did not recruit more numbers of participants because it was thought that estimating the grand trend in the emotional arousal changes of much larger numbers of participants (37 viewers in this study) with such a small number of participants would rather strengthen the practical usability of our method. That is, our study might suggest that the proposed method can effectively reduce the number of participants required for an evaluation of movies. Furthermore, given that the females are more sensitive to emotionally unpleasant stimuli than males (Bianchin and Angrilli, 2012), the proposed method has a potential to show improved performance with female participants. More studies are required to further validate the effectiveness of our method in practical movie evaluation applications.

**FIGURE 7:**
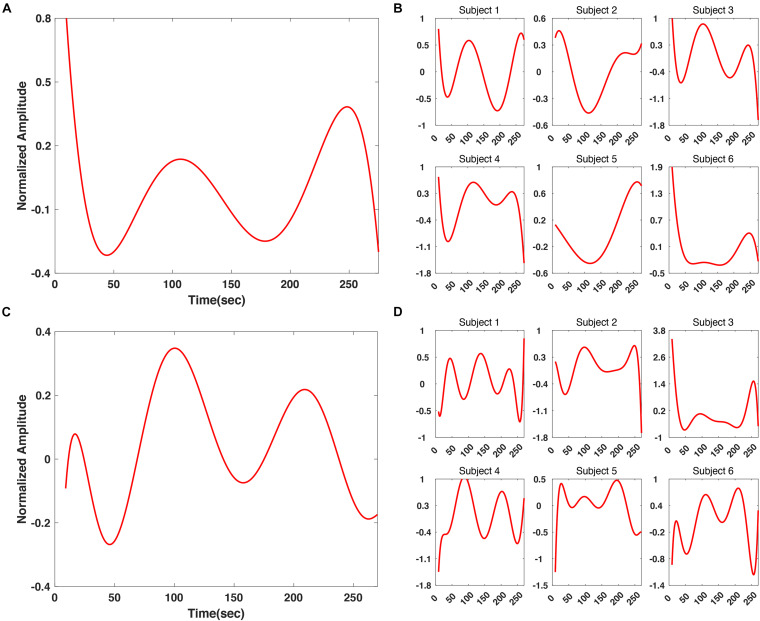
Comparison between grand averaged and individual polynomial regression results of SSVEP response at 6 Hz. **(A)** Grand averaged polynomial regression result for “fear” video. **(B)** Individual polynomial regression result for “fear” video. **(C)** Grand averaged polynomial regression result for “nervous” video. **(D)** Individual polynomial regression result for “nervous” video.

In addition, we applied z-score normalization to individual SSVEP responses before grand averaging, to prevent the averaged SSVEP results from being biased by data of certain participants. In Figure 7, the grand-averaged and individual SSVEP responses are presented. For the “fear” video data, the regression results for subject 1, 3, and 4 showed a good consistency with each other as well as the grand averaged results (Figures 7A,B). A tendency that the SSVEP responses rose at approximately 250 s was commonly observed in all subjects’ results. On the other hand, for the “nervous” video data, there was a good consistency among the regression results for all participants except for subject 3 (Figures 7C,D). Interestingly, a tendency showing high SSVEP responses at the beginning of the “fear” video was consistently observed for most of the participants, as shown in Figure 7B. It might originate from an “expectancy effect.” When the participants first realize that the video they are watching is a horror movie, their SSVEP response can be temporary increased because they generally predict fearful events to be happen, even though there is no actual fearful event in the scene. This enhancement of SSVEP response by “fear expectancy” was already reported in an MEG study (Moratti et al., 2006), in which the authors reported the SSVEP amplitude could be enhanced by merely an expectancy, through a fear-related motivated attention due to the activation of the defense system, even without an actual presentation of fearful stimulus.

In summary, we proposed a new method for estimating emotional arousal changes in a group of individuals during movie screening by overlaying SSVEP stimuli on the original video clips. Our experimental results agreed well with previous SSVEP studies that reported a positive correlation between the SSVEP amplitude and emotional arousal. This method might be used to track continuous emotional arousal changes in viewers during movie screening and to evaluate whether the movie has been edited well as intended by filmmakers.

## Data Availability Statement

The raw EEG datasets and survey results are available at the following link: https://figshare.com/s/745412a9047b478c3f81.

## Ethics Statement

The studies involving human participants were reviewed and approved by the institutional review board of Hanyang University. The patients/participants provided their written informed consent to participate in this study.

## Author Contributions

D-WK and C-HI designed the study. SP and D-WK conducted the experiment and data analyses. C-HH helped to perform the analyses through the constructive discussions. SP and C-HI wrote the manuscript. All authors reviewed and approved the final manuscript.

## Conflict of Interest

The authors declare that the research was conducted in the absence of any commercial or financial relationships that could be construed as a potential conflict of interest.

## Publisher’s Note

All claims expressed in this article are solely those of the authors and do not necessarily represent those of their affiliated organizations, or those of the publisher, the editors and the reviewers. Any product that may be evaluated in this article, or claim that may be made by its manufacturer, is not guaranteed or endorsed by the publisher.

## Footnotes

1
https://www.next-mind.com
